# *Caralluma fimbriata* extract activity involves the 5‐HT2c receptor in PWS *Snord116* deletion mouse model

**DOI:** 10.1002/brb3.1102

**Published:** 2018-10-23

**Authors:** Joanne L. Griggs, Michael L. Mathai, Puspha Sinnayah

**Affiliations:** ^1^ College of Health and Biomedicine Victoria University Melbourne Victoria Australia

**Keywords:** 5‐HT2c receptor, appetite signaling, cactus supplement, *Caralluma Fimbriata* extract, Prader–Willi syndrome (PWS), *snord116* deletion

## Abstract

**Introduction:**

In Prader–Willi syndrome (PWS), nonprotein coding small nucleolar (sno) RNAs are involved in the paternally deleted region of chromosome 15q11.2‐q13, which is believed to cause the hyperphagic phenotype of PWS. Central to this is *SnoRNA116*. The supplement *Caralluma fimbriata* extract (CFE) has been shown to decrease appetite behavior in some individuals with PWS. We therefore investigated the mechanism underpinning the effect of CFE on food intake in the *Snord116del* mouse. Experiments utilized appetite stimulants which included a 5‐hydroxytryptamine (5‐HT) 2c receptor antagonist (SB242084), as the 5‐HT2cR is implicated in central signaling of satiety.

**Methods:**

After 9‐week chronic CFE treatment (33 mg or 100 mg kg^−1^ day^−1^) or placebo, the 14‐week‐old *Snord116del* (SNO) and wild‐type mice (*n* = 72) were rotated through intraperitoneal injections of (a) isotonic saline; (b) 400 mg/kg of 2‐deoxyglucose (2DG) (glucose deprivation); (c) 100 mglkg beta‐mercaptoacetate (MA), fatty acid signaling; and (d) SB242084 (a selective 5HT2cR antagonist), with 5 days between reagents. Assessments of food intake were from baseline to 4 hr, followed by immunohistochemistry of neural activity utilizing c‐Fos, neuropeptide Y, and alpha‐melanocyte‐stimulating hormone within hypothalamic appetite pathways.

**Results:**

*Caralluma fimbriata* extract administration decreased food intake more strongly in the SNO100CFE group with significantly stimulated food intake demonstrated during coadministration with SB242084. Though stimulatory deprivation was expected to stimulate food intake, 2DG and MA resulted in lower intake in the *snord116del* mice compared to the WT animals (*p* = <0.001). Immunohistochemical mapping of hypothalamic neural activity was consistent with the behavioral studies.

**Conclusions:**

This study identifies a role for the 5‐HT2cR in CFE‐induced appetite suppression and significant stimulatory feeding disruptions in the *snord116del* mouse model.

## 1 INTRODUCTION

Prader–Willi syndrome (PWS) is a neurogenetic disorder characterized by neonatal hypotonia and failure to thrive. The syndrome follows a trajectory from these core criteria, through early childhood‐onset obesity to establishing hyperphagia (excessive appetite) and repetitive food‐focussed behaviors at the mean age of 8 years (Cassidy & Driscoll, 2009; McCandless, 2011). Typically, PWS is life‐threatening without routine management by dieting and supervision. This is mainly due to sleep apnea, choking, stomach necrosis, or obesity‐related complications (Stevenson et al., 2007). To date, pharmacological treatments for the suppression of appetite in PWS have shown minimal efficacy (Griggs, Sinnayah, & Mathai, 2015); however, interestingly the Indian cactus succulent *Caralluma fimbriata* (CFE) has demonstrated favorable modulation of hyperphagia and appetite behaviors in our clinical pilot study of children and adolescents with PWS (*n* = 15) (Griggs, Su, & Mathai, 2015).

Clinical studies of CFE's efficacy in non‐PWS obese adults have demonstrated suppression of appetite and reductions of waist circumference (Astell, Mathai, McAinch, Stathis, & Su, 2013; Kuriyan et al., 2007; Lawrence & Choudhary, 2004). Studies in animals report similar attenuation of food intake, and importantly comprehensive toxicity assessments assure the safety of treatment CFE (Odendaal et al., 2013; Sakore, Patil, & Surana, 2012). However, characterization of the mechanism of action of CFE remains hypothetical (Kamalakkannan, Rajendran, Venkatesh, Clayton, & Akbarsha, 2010, 2011 ; Komarnytsky, Esposito, Rathinasabapathy, Poulev, & Raskin, 2013; Rajendran et al., 2014). Observations in animals include improvements in the lipid profile and reduction in levels of leptin and/or blood glucose (Ambadasu, Dange, & Wali, 2013). Further, in mouse‐derived 3T3‐L1 cell lines, CFE has inhibited preadipocyte cell division during adipogenesis in a dose and time‐dependent manner (Kamalakkannan, Rajendran, Venkatesh, Clayton, & Akbarsha, 2010). It is proposed that CFE's mechanism of action involves the pregnane glycosides, both as an antihyperglycemic (Priya, Rajaram, & Sureshkumar, 2014) and as an antinociceptive (Rajendran et al., 2014) and that the various steroidal glycosides increase stimulation of the anorexigenic melanocortin pathway (Komarnytsky et al., 2013). These specific hypothalamic appetite pathways are believed to be disturbed due to genetic modifications, one specifically involved in modifying the genetic translation of the 5‐HT2c receptor.

Implicated in the PWS phenotype are five critically deleted protein coding paternal genes in the region on chromosome 15q11.2‐q13.1 *(MKRN3, MAGEL2, NECDIN, SNURF‐SNRPN*,* NPAP1/C15orf2)* and six nonprotein coding C/D box small nucleolar RNA (SnoRNA) genes or SNORDs: 107, 64, 108, 109, 116, and 115. The proximation of SnoRNAs’ regulation of premRNA splicing and methylation of targeted RNAs is not fully understood and not able to be approximated in animal models as little is known of their functional consequences. However, at this time the *SNORD116* is noted to be the most likely of the SnoRNA candidates pertaining to the severity of the syndrome in humans. In mice, deletion of SnoRNA116 influences serotonin or 5‐hydroxytryptamine (5HT)‐mediated behaviors and appetite modulation through neuropeptide Y (NPY) (Duker et al., 2010; Falaleeva, Surface, Shen, Grange, & Stamm, 2015; Gallagher, Pils, Albalwi, & Francke, 2002; McAllister, Whittington, & Holland, 2011; Qi et al., 2016; Zieba et al., 2015) also involved in homeostatic processes with the melanocortin pathway. We therefore chose to investigate regulation of food intake—due to CFE—in the Garvan S*nord116* deletion mouse model. This genetic deletion in the mouse’ chromosome 7C creates a hyperphagic phenotype (Duker et al., 2010; Kantor, Shemer, & Razin, 2006) which is relatively homologous with the human PWS paternally deleted chromosome 15. However, the animal does not become obese as is the case with all mouse models replicating genetic deletions from the PWS critical region (Bervini & Herzog, 2013; Golding, et al., 2017).

We hypothesized that CFE steroidal glycosides were involved in appetite suppression by enhancing 5‐HT2c receptor signaling (Canton et al., 1996; Doe et al., 2009; Falaleeva et al., 2015; Schellekens et al., 2015). Though typically 5‐HT or serotonin may be increased through pharmaceutical treatment, that is, selective serotonin reuptake inhibition (SSRI) (Griggs, Sinnayah, et al., 2015; Selikowitz, Sunman, & Wright, 1990); treatments of this nature require active 5‐HT receptors to release second messenger activity within the appetite pathways of the central nervous system (CNS). The literature describes 5‐HT2cR's anorexigenic receptor activity predominantly within the arcuate nucleus (ARC) of the hypothalamus (Reynolds, Hill, & Kirk, 2006). Moreover, in the ARC food intake may also be inhibited by plasma leptin increase in proopiomelanocortin (POMC) neurons coexpressing 5‐HT2cR mRNA (Khan, Gerasimidis, Edwards, & Shaikh, 2016 ^); (^Zhou et al., 2005). These anorexic signals further release a downstream melanocortin agonist, alpha‐melanocyte‐stimulating hormone (α‐MSH), via paraventricular (PVN) neural afferents of the hypothalamus. This signaling pathway activates inhibition via the melanocortin 4 receptor (MC4R) (Cone, 2005; Cowley, 2003; Ellacott, Halatchev, & Cone, 2006; Yosten & Samson, 2010) as a homeostatic response to balance food intake in opposition to the orexigenic NPY neuronal pathway. It is reported that hyperphagic feeding in the *Snord116del* mice is due to enhanced NPY activity (Bervini & Herzog, 2013; Zhang, Bouma, McClellan, & Tobet, 2012); we therefore targeted the melanocortin appetite pathway (Komarnytsky et al., 2013) via the 5‐HT2c receptor to firstly determine homeostatic balance with NPY (Heisler et al., 2006; Zhou et al., 2005) and to discover whether CFE signaling involved this receptor. As this receptor has been reported to be disrupted in humans with PWS (Angulo, Butler, & Cataletto, 2015; Isles, 2017; Kishore & Stamm, 2006; Stamm, Gruber, Rabchevsky, & Emeson, 2017), it is important to understand whether CFE's action involves this pathway. The therefore utilized an appetite stimulant 5‐HT2cR antagonist, (iv) SB 242084, which was expected to stimulate feeding by inhibiting serotonin transmission (Kennett et al., 1997 ^); (^Lam et al., 2007).

We were also keen to elucidate whether any hunger experienced by the *Snord116del* mouse was due to CFE or due to metabolic alterations regarding carbohydrates, fatty acids, or malabsorption of food. It is well known that ingestion of carbohydrates or dietary fatty acids increases blood levels of glucose or lipids, which in turn modify appetite signaling in the hypothalamus. Past research on CFE has demonstrated that pregnane glycosides (the proposed active constituent of CFE) alter lipid metabolism and inhibit fatty acid biosynthesis (Selikowitz et al., 1990). To this end, we utilized acute stimulation of food intake through administration of 2‐deoxy‐D‐glucose (2DG), which stimulates glucoprivic feeding and sodium mercaptoacetate (MA), which stimulates lipoprivic feeding through either inhibition of the availability of glucose or fatty acids as metabolic fuels (Li, Wiater, Wang, Wank, & Ritter, 2016; Ritter & Taylor, 1990). Administration of 2DG or MA has been utilized to stimulate food intake and determine specific areas of neuronal activation within the brain in rodents (Ritter & Taylor, 1990). These variables were important to consider when determining if observed short‐term appetite results were associated with the metabolic pathway, treatment, or the mouse strain. The full first round of stimulatory experiments was immediately followed by acute stimulatory experiments with 2DG for immunohistochemistry investigations of hypothalamic cell activity within the CNS in the same animals.

## 2 METHODS

### 2.1 Animals

The *Snord116del* mouse strain homozygote (Ding et al., 2008) mating pairs and wild‐type (WT) control homozygote mating pairs (bred from the same original C57BL/6 laboratory stock) (Purtell, Aepler, Qi, Campbell, & Herzog, 2017) were generously gifted to our study by Herbert Herzog of the Garvan Institute. These animals were transported to the Florey Neuroscience Institute (FNI), Melbourne, Australia, in accordance with relevant guidelines under ethics approval 14‐081 FINMH. The strain was obtained with permission from Jackson Laboratory (Bar Harbor, ME, USA) C57BL/6(Cg)‐Snord116tm1Uta/J; Stock No: 008118) and was originally bred at ABR (Australian Bio‐Resources Pty ltd., Moss Vale, NSW, Australia). The colony raised 72 mice (*Snord116del*/SNO × 36 18 male (M) & 18 female (F), WT × 36—18 M and 18F), bred within 7 weeks of each other. All mice stayed with their mothers until weaning at 4 weeks of age. Due to good numbers per litter, this study was able to utilize only first‐generation animals from the mating pairs for the experimental protocol. No pair had more than two litters within the timeline and these were then divided into six groups of 12, and single‐housed in standard conditions in a temperature‐controlled (21°C) mouse facility with a 12‐hr light: dark cycle (lights on 0600–1800 hr). The groups were randomized by weight, parentage, and sex. At the time of the experiments, the 3 × *Snord116del* groups were 5% lighter on average, with animals’ body weight ranging from *Snord116del*/SNO: 17.6–23.8 g and WT: 18.2–25.8 g at 14 weeks.

### 2.2 Treatments

From 6 weeks of age, the strain groups received basic chow and either a daily treatment (dose per weight) of standardized CFE powdered extract (CFE 33 or 100 mg/kg) or a placebo (PLAC) (200 mg/kg maltodextrin and 50 mg cabbage leaf), a similarly mild‐bitter tasting powder (SNO × 12/WT × 12 (Male (M) × 6 & Female (F) × 6—CFE100 mg/kg), SNO × 12/WT × 12 (M × 6 & F × 6—CFE33 mg/kg) and SNO × 12/WT × 12 (M × 6 & F × 6—PLAC). Both treatments were dissolved in jelly and ingested over 9 weeks (wks) before acute stimulation of food intake with (a) administration of isotonic saline (SAL); (b) administration of 2DG; and (c) MA. To make the jelly daily, CFE and PLAC were dissolved in 25 ml of water and added to 125 ml of gelatine (Davis Gelatine, GELITA Australia Pty. Ltd) (Purtell et al., 2017). Each treatment contained 2% saccharin (0.05 mg = 2%) as a heat stable low‐calorie sweetener that ensured voluntary ingestion. Visual confirmation of all jelly ingestion was made as well as daily measurements of body weight and food intake. Water was provided ad libitum.

### 2.3 Appetite stimulation

At 14 wks of age during the 9th week of administration, appetite stimulants were administered by intraperitoneal (i.p) injection, in the order a–d, with 4to 5‐day break between each experimental protocol. The experimental reagents were (a) SAL—isotonic saline, utilized as a control; (b) 2DG—2‐deoxyglucose (400 mg/kg, Sigma‐Aldrich 2‐deoxy‐D‐glucose, D83755G), prompting glucose deprivation; (c) MA—beta‐mercaptoacetate (100 mg/kg, Sigma‐Aldrich, methyl thioglycolate 108995‐100G), stimulating fatty acid signaling, and (d) SB 242084—5‐HT2cR antagonist (1 mg/kg, Sigma‐Aldrich, SB 242084 dihydrochloride hydrate, S8061‐5 mg), prompting an orexigenic response.

On the morning of each protocol either a, b, c, or d, the animals’ weights were recorded for reagent volume and daily treatment CFE or PLAC was confirmed as ingested. The timeline for the tests incorporated 2 hr of fasting preinjection and 4 hr of observation postinjection. Food and water were removed (2 hr) before baseline. At baseline (14.30 p.m.), all groups (SNO100CFE; SNO33CFE; SNOPLAC or WT100CFE; WT33CFE and WTPLAC) received that day's protocol reagent (SAL; 2DG; MA or SB242084 in 1 ml saline solution) by i.p. injection at the specified volume for each animal's weight. Measured data involved the amount of basic chow eaten and water consumed by each animal after 4 hr, which included 1 hr into the dark cycle, where natural feeding was expected. The administration of treatments, stimulants, and measurements of food and water followed an identical ordered cycle in all 72 first‐generation animals.

### 2.4 Immunohistochemistry

At 17 wks of age, after statistical analysis of the stimulant experiments, the mice ingesting CFE at the highest dose CFE100CFE or PLAC (SNO100CFE; SNOPLAC; WT100CFE or WTPLAC, *n* = 48) were randomly allocated to either 2DG acute stimulant or control SAL groups for immunohistochemistry experimentation. At 90 min, eight randomly chosen mice per day were weighed to the nearest 0.1 gram to determine the reagent volume and were given an i.p. injection of SAL or 2DG before. The animals were then timed for perfusion, for which they were given an i.p. injection of sodium pentobarbitone (dose—80 mg/kg). They were perfused transcardially by normal saline and paraformaldehyde (PFA) (4%) in 0.1 M phosphate buffer (PB) (pH7.2). The brains were removed and cryoprotected for storage at (20°C) until immunohistochemistry processing by immersion in 30% sucrose by weight (w/v) in 0.1 M PB for 72 hr followed by freezing in isopentane. Immunohistochemistry processes were carried out by the Melbourne Brain Centre, Victoria, Australia. Free‐floating coronal sections of 40 µm thickness were cut on a Leica Microsystems CM1850 cryostat relative to the specified area of interest (AOI)from Bregma 1.10 mm to −2.92 mm (striatum, hypothalamus, midbrain) of the mouse brain: a) ARC: arcuate nucleus of the hypothalamus b) PVN: paraventricular nucleus of the hypothalamus. The sections were placed into cryoprotectant solution (30% ethylene glycol and 15% sucrose in PB) and stored at −20°C prior to staining.

### 2.5 Triple labeled Immunofluorescence

Primary antibodies were diluted in antibody diluent (1% normal donkey serum (NDS) and 1% Triton X‐100 in PB) as follows: rabbit anti‐c‐Fos primary 1:2000, anti‐NPY 1:1,000, (Abcam, anti‐NPY antibody: ab112473 NPY, mouse monoclonal) and anti‐MSH 1:10,000 (Merc‐Millipore, anti‐MSH α‐antibody). Sections were incubated with primary antibodies over two nights at 4°C with agitation. Before staining, the sliced sections were washed in PB (2 × 10 min) and were blocked for 30 min at room temperature with agitation in 10% NDS and 1% Triton X‐100 (Sigma‐Aldrich) in PB. Secondary antibodies were diluted in antibody diluent at 1:400 NPY—donkey anti‐mouse/rabbit 488 (green) (Alexa Fluor® 488 AffiniPure donkey anti‐mouse) IgG (H + L) (Luo et al., 2015), and α‐MSH: donkey anti‐rabbit IgG (H + L), secondary antibody (red) (Alexa Fluor® 594 conjugate a21207) (Zhang, 2011), and c‐Fos donkey anti‐sheep 647 (purple) (Alexa Fluor® 647) (Purkartova et al., 2014). Sections were incubated with secondary antibodies (3 hr) at room temperature. Sections were washed and mounted on Superfrost Plus slides (Hurst Scientific) in mounting medium. Images of the mouse brain sections were obtained at 40X magnification using a Nikon E400 confocal microscope.

### 2.6 Statistical analysis

Results were analyzed using SPSS version 23.0 for Windows. All data were determined as normally distributed with *p *≤* *0.05 as significant. Analysis of food intake, thirst (not shown), and totals of c‐Fos labeled neurons were organized into mean ± *SD* per group. The results involved food ingested by strain × 2; treatment × 3; and stimulant × 4 in groups (*n* = 12). Effect sizes were analyzed by two‐way MANOVA for significance, followed by a repeated‐measures ANOVA for univariate measures, with Bonferroni corrections for multiple comparisons. This determined the significance of the reagents against both strain and treatment as between‐group factors of variance. ANOVAs were followed by unpaired *t* tests analyzing stimulating reagents against SAL control for within group factors of variance.

Labeled cell numbers were analyzed by a two‐way ANOVA. Normality with no missing values was met in the groups (*n* = 5), with two dependent variables of strain (*Snord116del*/SNO and WT), two dependent variables of treatment (100 CFE/kg/day & PLAC), and two dependent variables of stimulation (SAL and 2DG). There were three independent variables of labeled channel activity, FITC (488): NPY; AL × 594 (561): α‐MSH and Cy5 (640): c‐Fos × 2‐3 slides per AOI. The results were defined with strain, treatment, and stimulation as factors of variance. This was followed by post hoc, Tukey's tests to specifically pinpoint significance related to the eight group variations (*n* = 5 mice × 8 groups: ARC × 2‐3 sections per mouse, PVN × 2‐3 sections per mouse); per image (*n* × 7; c‐Fos; NPY/c‐Fos; α‐MSH/c‐Fos; NPY/α‐MSH; and NPY/α‐MSH/c‐Fos).

## 3 RESULTS

### 3.1 Appetite stimulation

The stimulation results determined complex significant differences in food intake due to treatments, stimulants, and strain. The lowest food intake recorded over the 4 hr within *Snord116del* animals was in the 100CFE group during 2DG stimulation (Table 1) and for the WT strain, during MA stimulation in the 100CFE animals. Under SAL control conditions, the *Snord116del* strain demonstrated hyperphagia, eating more than the WT animals: *Snord116del*/SNO (*n* = 36) 1.39 ± 0.32 g; WT (*n* = 36) 0.89 ± 0.32 g (*p *=* *0.007). Though their food intake was higher, there was still a small significant dose response in reduced food intake due to CFE's dose, SNO100CFE animals, against the lower dose 33CFE, SAL—SNO100CFE 1.27 ± 0.27 g; SNO33CFE 1.47 ± 0.23 g (*p *=* *0.03). In comparison under basal conditions, the WT mice demonstrated no significant alterations between the treatment groups SAL—WT100CFE 0.81 ± 0.27 g; WT33CFE 0.94 ± 0.30 g (NS, *p *=* *0.32).

**Table 1:** Saline comparison of food and water intake after stimulants—2DG, MA, and the 5HT2c receptor antagonist SB 242084

Comparisons SAL	SAL		2DG			MA			SB 242054		
	Mean	*SD*	Mean	*SD*	*p*	Mean	*SD*	*p*	Mean	*SD*	*p*
FOOD *Snord116del* (g)											
100CFE (*n* = 12)	1.21	0.27	0.78	0.37	<0.001**	1.00	0.23	0.02*	1.46	0.38	0.11
33CFE (*n* = 12)	1.47	0.23	0.98	0.29	<0.001**	1.05	0.37	0.003**	1.60	0.64	0.51
PLAC (*n* = 12)	1.46	0.41	1.01	0.26	0.003**	0.94	0.31	0.003**	1.55	0.47	0.63
FOOD WT (g)											
100CFE (*n* = 12)	0.81	0.27	1.19	0.18	<0.001**	0.78	0.37	0.14	1.61	0.61	<0.001**
33CFE (*n* = 12)	0.94	0.30	1.36	0.30	0.002**	0.98	0.29	0.55	1.79	0.7	<0.001**
PLAC (*n* = 12)	0.91	0.39	1.56	0.67	0.008**	1.01	0.26	0.47	1.25	0.44	0.06

Note

Unpaired *t* tests with the mean and standard deviation (SD) of food in grams (g) ingested over 4hrs from baseline, in the Snord116del mouse model and C57BL/6 wild type (WT) strain. Measurement after intraperitoneal (i.p.) injections of control saline (SAL) or stimulants: 2‐deoxy‐glucose (2DG), (400mg/kg, 10mg = 25g mouse), beta – mercaptoacetate (MA) (100mg/kg, 2.5mg = 25g mouse), or the 5‐HT2cR antagonist SB242804 (1.0mg/kg, 025mg= 25g mouse). The randomized factors were treatment: extract of Caralluma fimbriata (CFE) x 2 doses (100mg/kg/d & 33mg/kg) or placebo of maltodextrin/cabbage leaf (PLAC). The level of significance is reported as p = 0.05* and p = 0.001**.

Unusually even though the *snord116 deletion* caused hyperphagia; during the acute stimulation tests, the *Snord116del* mice experienced significant inhibitions of food intake.

Within strain comparisons of glucoprivic and lipoprivic food intake demonstrated that though i.p injections of 2DG stimulated feeding in the WT animals, 2DG did not stimulate feeding in the *Snord116del* mice (Table 1), 2DG: SNO100CFE 0.78 ± 0.37 g; WT100CFE 1.19 ± 0.18 g, (*p *=* *0.002); SNO33CFE 0.98 ± 0.29 g; WT33CFE 1.36 ± 0.30 g (*p *= <0.001); and 2DG:—SNOPLAC 1.01 ± 0.26 g; WTPLAC 1.56 ± 0.67 g (*p *=* *0.01). In fact, the *Snord116del* strains food intake were indicative of signaling disruptions with significant inhibition of food intake in response to 2DG, SAL—SNO100CFE 1.27 ± 0.27 g in comparison with 2DG—SNO100CFE 0.78 ± 0.37 g (*p *=* *0.002); SAL—SNO33CFE 1.47 ± 0.23 g; 2DG—SNO33CFE 0.98 ± 0.29 g (*p *= <0.001). Further, the results suggested this inhibited stimulation was due to the *Snord116* deletion and not treatment with CFE as reduced food intake in the *Snord116del* strain during glucose deprivation was similar in the PLAC group, 2DG—SNOPLAC 1.01 ± 0.26 g; SAL—SNOPLAC 1.46 ± 0.41 g (*p *=* *0.003). In the WT strain under 2DG stimulated deprivation treatment, CFE only resulted in a trend in lessening food intake 2DG:—WT100CFE 1.19 ± 0.18 g; WT33CFE 1.36 ± 0.30 g; WTPLAC 1.56 ± 0.67 g (NS, *p *=* *0.08).

**Figure 1:**
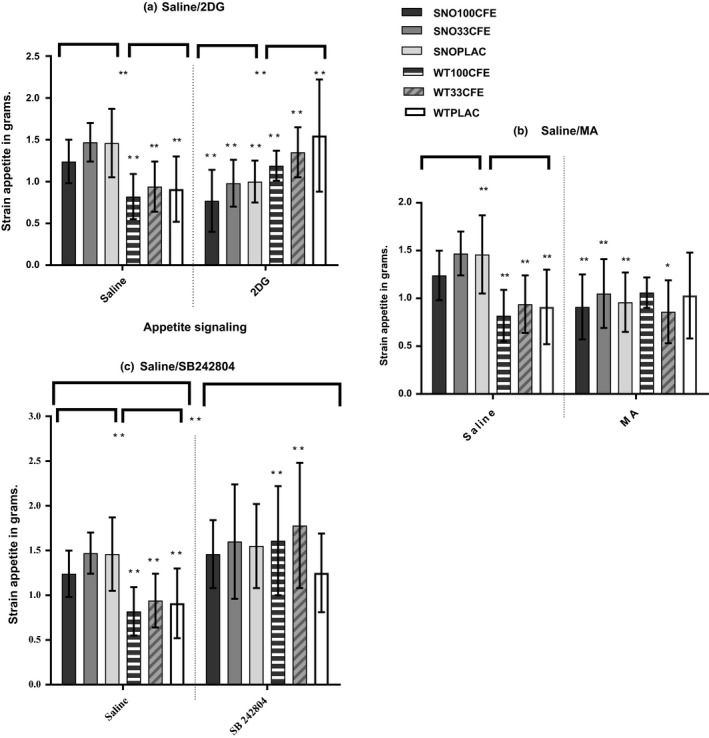
Appetite Stimulant Histograms. Figure 1 depicts the univariate between‐subject results for appetite signaling tests with SAL—saline control, mean and *SD*—standard deviation of food ingested in grams in comparison with the appetite signaling reagents 2DG—2‐deoxyglucose, MA—beta‐mercaptoacetate or the 5‐HT2c antagonist SB242804. The results present pairwise comparisons for food ingested over four hours, with significance set as Pillai's trace Sig value, *p *=* *≤0.05, plus *t* tests between three chronic treatment groups saline versus appetite signaling. The animal models were the *Snord116del* (SNO) and wild‐type (WT) strains. All animals were ingesting a chronic treatment of either CFE—*Caralluma fimbriata* extract, at one of two doses 100 mg/kg/d or 33 mg/kg/d or PLAC—placebo of maltodextrin/cabbage leaf [SNO: *n* = 36; WT: *n* = 36: (100CFE/M: *n* = 6 and F: *n* = 6; 33CFE/M: *n* = 6 and F: *n* = 6; and PLAC/M: *n* = 6 and F: *n* = 6)]

MA's stimulatory effect was minimal in all groups though there were also some unexpected strain comparisons (Table 1 and Figure 1). Once again unexpectedly, stimulation inhibited food intake in the *Snord116del* strain. MA fatty acid deprivation resulted in significant differences between strain's treatment groups (Table 1).

The major significant interactions within strains in food intake were during administration of the 5HT2c receptor antagonist SB 242084. A higher food intake was measured in animals that had ingested CFE over the prior 9 weeks, as compared to PLAC. The WT animals demonstrated significant differences in food intake especially due to administration of CFE, SAL—WT100CFE 0.81 ± 0.27; SB 242084 WT100CFE 1.61 g ± 0.61 g (*p* ≤ 0.001), SAL, WT33CFE 0.94 ± 0.30; SB 242084 WT33CFE 1.79 g ± 0.70 g (*p*≤ 0.001). The PLAC group demonstrated less increase in food intake (NS). The strongest increase in food intake within the *Snord116del* strain was in the SNO100CFE due to SB 242084 antagonism. However, this result was not significant as the *Snord116del* strain also exhibited hyperphagia under control conditions, SAL:—SNO100CFE 1.27 ± 0.27 g; SB 242084 SNO100CFE 1.46 g ± 0.38 g (NS, *p *=* *0.11).

Over all experiments, the most significant difference in food intake was seen in the WT animals during SB 242084 antagonist administration, SAL—WT33CFE 0.94 ± 0.30; SB 242084 WT33CFE 1.79 g ± 0.70 g (*p *≤ 0.001) (Table 1). The 5‐HT2cR antagonist SB 242084 was the most powerful stimulant in both strains and all treatment groups. SB 242084 enhanced the feeding for all groups, including the PLAC groups. These experiments suggest the 5‐HT2c receptor is involved in the mechanistic activity of CFE. In the WT animals, food intake was significantly increased in the 100CFE and 33CFE treatment groups, compared to the PLAC group, SB 242084—WT100CFE 1.61 g ± 0.61 g; WTPLAC 1.25 g ± 0.44 g (*p *=* *0.02), and SB 242084—WT33CFE 1.79 g ± 0.70 g, WTPLAC 1.25 g ± 0.44 g (*p *=* *0.04).

### 3.2 Immunohistochemistry

**Figure 2:**
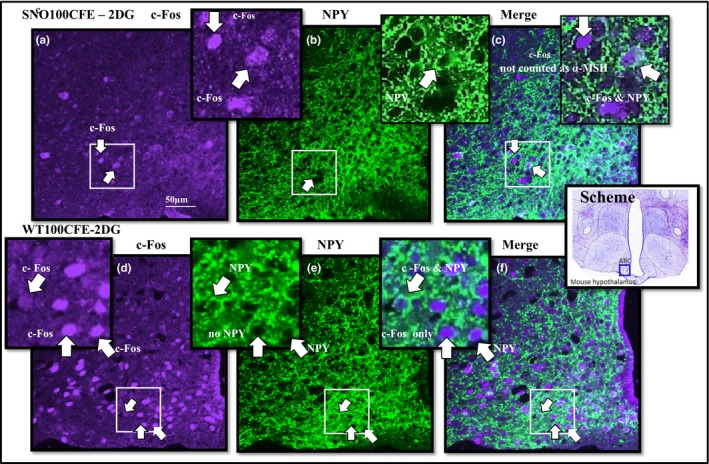
Immunohistochemistry comparison of strain c‐Fos and NPY cell population. Colocalization of neuropeptide and activity: color image (purple) c‐Fos—Fos‐like early gene expression (green); NPY—neuropeptide Y and c‐Fos in brain slices from the ARC—arcuate nucleus of the hypothalamus in SNO (*n* = 5)—representative of *Snord116del* mice and in WT (*n* = 5)—wild‐type control, ingesting chronic treatment 100CFE—*Caralluma fimbriata* extract, at 100 mg/kg/d or PLAC—placebo 200 mg maltodextrin and 50 mg cabbage leaf, with food intake stimulant, signaling reagents, 2DG—2‐deoxyglucose, induced by i.p. injection (400 mg/kg, 10 mg =25 g mouse), in comparison with the control SAL—i.p. injection of isotonic saline

**Figure 3:**
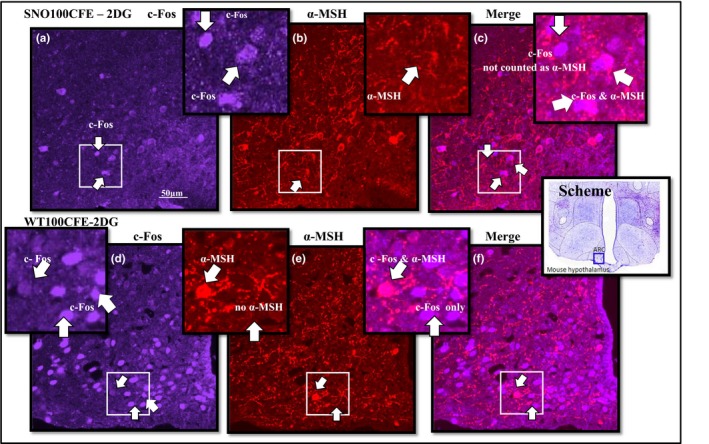
Immunohistochemistry comparison of strain c‐Fos and α‐MSH cell population. Colocalization of neuropeptide and activity: Color image (purple) c‐Fos—Fos‐like early gene expression (red); α‐MSH—alpha‐melanocyte‐stimulating hormone; and C‐Fos in brain slices from the ARC—arcuate nucleus of the hypothalamus in SNO (*n* = 5)—representative of *Snord116del* mice and in WT (*n* = 5)—wild‐type control, ingesting chronic treatment 100CFE—*Caralluma fimbriata* extract, at 100 mg/kg/d or PLAC—placebo 200 mg maltodextrin and 50 mg cabbage leaf, with food intake stimulant, signaling reagents, 2DG—2‐deoxyglucose, induced by i.p. injection (400 mg/kg, 10 mg =25 g mouse), in comparison with the control SAL—i.p. injection of isotonic saline

In the ARC, there was a significant difference in NPY and c‐Fos dual‐labeled neurones between strains in response to 2DG stimulation, NPY—SNOPLAC 21.0 ± 15.93; WTPLAC 159.8 ± 65.53 (*p *= <0.001) (Figure 2). These results were supported by coexpression of c‐Fos and NPY in the PVN, which demonstrated significant differences between the strains in response to 2DG—SNOPLAC 51.0 ± 15.84; WTPLAC 138.2 ± 49.17 (*p *=* *0.005) and the 2DG NPY—SNO‐PLAC 24.0 ± 12.94; WT‐PLAC 76 ± 18.07 (*p *= <0.001). Comparisons of activity through α‐MSH and c‐Fos dual‐labeled neurones indicated the significant differences between strains. The highest WT satiety signaling activity, due to treatment CFE, was in the ARC of the control SAL—WT100CFE and in the *Snord116del* strain was due to 2DG stimulation, 2DG—SNO100CFE (Figure 3 and Table 2). The SNOPLAC‐SAL controls had the lowest α‐MSH colocalization to c‐Fos (Table 2).

**Table 2:** Pairwise comparisons of immunohistochemistry cell counts, c‐Fos, NPY, and α‐MSH activity in the *Snord116 del* and wild‐type mouse brain hypothalamus

ARC treatment groups & stimulant/control	c‐Fos		c‐Fos pairwise comparison	*p* value	NPY + c‐Fos		NPY + c‐Fos pairwise comparisons	*p* value	α‐MSH + C‐Fos		α‐MSH + c‐Fos pairwise comparisons
c‐Fos	Mean	*SD*			Mean	*SD*			Mean	*SD*	
SNO‐100CFE‐2DG	88.2	±19.18	All SNO groups	(NS)	51.0	±37.51	All SNO groups WT‐PLAC‐2DG	(NS) 0.005**	28.2	±10.47	(NS)
SNO‐100CFE‐SAL	118.8	±28.41	SNO‐PLAC‐SAL	(NS)	77.2	±43.82	All other groups	(NS)	16.2	±15.99	(NS)
SNO‐PLAC‐2DG	49.6	±16.68	WT‐PLAC‐2DG	<0.001**	21.0	±15.93	WT‐PLAC‐2DG	<0.001**	16.8	±12.11	(NS)
SNO‐PLAC‐SAL	51	±15.84	WT‐PLAC‐SAL	0.02*	24.0	±12.94	All SNO groups WT‐PLAC‐2DG	(NS) <0.001**	3.8	±3.11	(NS)
WT‐100CFE‐2DG	146.2	±43.56	WT‐PLAC‐2DG	0.04*	109.2	±42.84	SNO‐PLAC‐2DG	0.04*	20	±14.07	(NS)
WT‐100CFE‐SAL	138.6	±24.13	SNO‐PLAC‐SAL	0.006**	62.6	±33.9	WT‐PLAC‐2DG	0.02*	30.8	±24.24	(NS)
WT‐PLAC‐2DG	218.4	±55.38	All groups	0.04* to <0.001**	159.8	±65.53	Sig. as above. SNO‐100CFE‐ SAL WT‐100CFE‐2DG WT‐PLAC‐SAL	0.01* to <0.001** (NS) (NS) (NS)	20.8	±11.88	(NS)
WT‐PLAC‐SAL	128.6	±40.12	WT‐PLAC‐2DG	0.005**	98.6	±47.41		(NS)	17.6	± 9.15	(NS)

Note

ANOVA post hoc pairwise comparisons with estimated marginal means and *SD*—standard deviation. Results of immunohistochemistry cell counts for c‐Fos—Fos‐like early gene expression, NPY: neuropeptide Y, and α‐MSH: alpha‐melanocyte‐stimulating hormone, in brain slices from the ARC—arcuate nucleus of the hypothalamus of two mouse strains: SNO—Garvan *Snord116del* (*n* = 5) and WT—C57BL/6 wild type (*n* = 5) per group. Animals were ingesting either chronic treatment 100CFE—*Caralluma fimbriata* extract, at 100 mg/kg/d or PLAC—placebo of maltodextrin/cabbage leaf with appetite signaling reagents, 2DG—2‐deoxyglucose compared to the control of SAL—saline.

## 4 DISCUSSION

CFE administration induced strain‐specific differences in food intake in response to acute stimuli. In the *Snord116del* mice, the lowest food intake was in the SNO100CFE group under all conditions (Table 1). There were also significant differences between the treatment groups in the WT strain due to CFE, during stimulation, that is, lower during MA stimulation and significantly higher during stimulation by SB 242084. The SB 242084 antagonist significantly increased stimulated food intake in all the WT treatment groups. The significant increases in the CFE groups and less increase in the PLAC group suggest that CFE interacts with activating signaling through the 5‐HT2c receptor. It therefore follows that CFE's attenuation of food intake involves potential downstream α‐MSH PVN signaling, though this would need further research (Zhou et al., 2005). The antagonist was expected to suppress all serotonin signaling through this receptor. Though there is no significant difference in the *Snord116del* mouse model's comparisons (SAL vs. SB 242084), this may be due to an overall functional inactivity. Clearly, it is a limitation of this study that this behavioral observation was not able to be tested by immunohistochemistry.

In the control SAL groups, it is important to address the observation that the *Snord116del* mice ate more than the WT during the 4‐hr food intake tests. This result was consistent with the hyperphagic phenotype of the *Snord116del* mouse model characterizations in the literature (Qi et al., 2016); (Overstreet‐Wadiche, Bensen, & Westbrook, 2006). Also notable are the complexities in reduced food intake involving administration of 2DG and MA in the *Snord116del* mice (Table 1). When deprived of glucose, the natural homeostatic response in a mouse is to reduce the amount of energy utilized and to consume food (Herzog, 2012). However, the *Snord116del* animals demonstrated unexpected and distinct behavioral effects during stimulated glucose deprivation. The most common observation was of a curled still posture as though the mice were conserving energy. Further, this was most strongly observed in the *Snord116del* mice ingesting 100CFE. Overall, this behavioral observation may be due to the *Snord116del* mouse model's lean phenotype and is consistent with another report of lower 5‐hr fasting glucose levels (Qi et al., 2016). Yet, further study may show that the *Snord116del's* conserving of energy is due to disrupted signaling specifically necessary for glucoprivic feeding and that CFE exaggerates the attenuation of food intake related to glucose homeostasis. Further, though MA stimulation did not significantly increase food intake in the WT groups, it did significantly lower food intake in all *Snord116del* groups against SAL. Though it is unlikely that glucoprivic or lipoprivic signals contribute to food intake during daily events in humans, research into glucose deprivation or *β‐*oxidation of fatty acids may be worth following through associated with both CFE and the *SNORD116* deletion in humans.

The SNO‐2DG activation of NPY neurones as indicated by c‐Fos and NPY, in both the CFE and PLAC groups, was consistent with the lower food intake. Regarding cell counts, it is apparent that 2DG stimulation resulted in lower c‐Fos and NPY signaling in the SNO‐2DG hypothalamus in both ARC and PVN, especially in comparison with the higher c‐Fos and NPY dual‐labeled counts in the WT‐2DG groups. Within all groups, the highest c‐Fos and NPY activity was consistent with the behavioral food intake in the WTPLAC animals. In contrast, the SNOPLAC group had a lower number of activated NPY neurones compared to the *Snord116del* group treated with CFE. This was in response to both 2DG and the control SAL, even though the SNOPLAC group ate more food. This is not as consistent though it is possible that the strength of α‐MSH activity in the ARC may have counteracted the excitatory activity of c‐Fos and NPY. Though not significant, in all treatment groups c‐Fos and α‐MSH activity was strongest after CFE treatment (Table 2). Research is necessary to verify signaling downstream through α‐MSH pathways linked with CFE's proposed mechanism of activity via the 5‐HT2c receptor. This may involve studying the effect of CFE on SAL scores in mice with the 5‐HT2c receptor knocked out, as compared to WT mice.

Within all groups and conditions, the strongest food intake activity was in the WTPLAC animals, which under 2DG stimuli was consistent with the increased activity of NPY neurones. The SNO‐2DG cell counts in both the CFE and PLAC groups confirmed the lower intake in food. It is possible that 2DG stimulation in the *Snord116del* mice interrupted the NPY activity in both ARC and PVN, especially in comparison with the higher NPY activity observed in the WT‐2DG groups. Unexpectedly, the SNOPLAC groups had a lower number of activated c‐Fos and NPY neurones compared to the *Snord116del* animals ingesting treatment CFE. Even though this difference was observed in both 2DG and the control SAL groups, the strength of the α‐MSH activity in the ARC may have gone some way to explain the behavioral inhibition of the excitatory activity of NPY in the SNO100CFE group.

Our research will now investigate increased signaling of satiety through downstream CNS pathways due to CFE and most importantly clinical trials research in humans with PWS—investigating the efficacy of CFE treatment—may ultimately need to involve incorporating dose escalation of CFE (Griggs, Su, et al., 2015). Observations may include 5‐HT2c receptor lymphocyte collection, to determine enhanced transcription, translation, or activity of fully functioning 5‐HT2c receptors.

## 5 CONCLUSION

The results of the experiments investigating CFE determined an involvement of serotonin via the 5‐HT2cR in the inhibitory effect of CFE on food appetite. This study also determined that CFE treatment alters food intake in the *Snord116del* animals, though hyperphagia is still present compared to the WT controls. Importantly, although glucoprivic and lipoprivic stimuli would be expected to increase food intake, in the *Snord116del* strain, the administration of reagents 2DG and MA resulted in reduced stimulation of food intake. This was especially strong in the group ingesting CFE, compared to the stimulated feeding in the WT animals. Immunohistochemical mapping of neuronal activation was consistent with the feeding behavior.
